# Mgs1 function at G-quadruplex structures during DNA replication

**DOI:** 10.1007/s00294-020-01128-1

**Published:** 2020-11-25

**Authors:** Katrin Paeschke, Peter Burkovics

**Affiliations:** 1 Department of Oncology, Hematology and Rheumatology, University Hospital Bonn, Bonn, Germany; 2 Institute of Genetics, Biological Research Centre, Szeged, Hungary

**Keywords:** G-quadruplex, Replication, Mgs1, Genome stability

## Abstract

The coordinated action of DNA polymerases and DNA helicases is essential at genomic sites that are hard to replicate. Among these are sites that harbour G-quadruplex DNA structures (G4). G4s are stable alternative DNA structures, which have been implicated to be involved in important cellular processes like the regulation of gene expression or telomere maintenance. G4 structures were shown to hinder replication fork progression and cause genomic deletions, mutations and recombination events. Many helicases unwind G4 structures and preserve genome stability, but a detailed understanding of G4 replication and the re-start of stalled replication forks around formed G4 structures is not clear, yet. In our recent study, we identified that Mgs1 preferentially binds to G4 DNA structures in vitro and is associated with putative G4-forming chromosomal regions in vivo. Mgs1 binding to G4 motifs in vivo is partially dependent on the helicase Pif1. Pif1 is the major G4-unwinding helicase in *S. cerevisiae*. In the absence of Mgs1, we determined elevated gross chromosomal rearrangement (GCR) rates in yeast, similar to Pif1 deletion. Here, we highlight the recent findings and set these into context with a new mechanistic model. We propose that Mgs1's functions support DNA replication at G4-forming regions.

## Introduction

Precise replication of the genome is essential for most eukaryotic cells, as it determines the fate of the daughter cells. Failure of precise replication can lead to genome instability, cancerous transformation or apoptosis. The continuous movement of the replication fork is often stalled by various obstacles, like different hard-to-replicate alterations on the template DNA strand, DNA-bound protein complexes, DNA damage or stable secondary structures (Aguilera and Garcia-Muse 2013). The stalled replication fork can be rescued by different pathways, including a direct bypass of the lesion or template switching where the newly synthesised DNA strand serves as a template (Unk et al. 2010). Post-translational modifications of PCNA, a homotrimer ring-like protein, regulates the re-start/repair of the stalled fork (Moldovan et al. 2007; Arbel et al. 2020; Ripley et al. 2020). Ubiquitylation of PCNA by the Rad6/Rad18 complex activates the DNA damage tolerance pathway (Hoege et al. 2002) whereas PCNA SUMOylation inhibits unwanted recombination events at the stalled fork (Papouli et al. 2005; Pfander et al. 2005; Motegi et al. 2006; Burkovics et al. 2013).

DNA can adopt alternative secondary structures in addition to the standard B-DNA conformation. The G-quadruplex (G4) structure is a stable, alternative DNA or RNA structure, which can form in specific guanine-rich sequences. The core of this structure is a G-quartet: four guanines form a planar cyclic arrangement which is stabilized by Hoogsteen base pairing. Stacking of G-quartets leads to a higher ordered structure that is stabilized by monovalent cations, most frequently potassium (Lipps and Rhodes 2009; Bochman et al. 2012; Chen and Yang 2012). Genomic regions with a high potential to fold into G4 structures can be determined experimentally as well as computationally (Huppert and Balasubramanian 2007; Todd and Neidle 2011; Hansel-Hertsch et al. 2017; Marsico et al. 2019). G4s were identified at telomeres and many endogenous sites (intrachromosomally) in all eukaryotic cells tested so far. It is assumed that depending on the cell cycle, developmental phases, exogenous or endogenous stimuli different G4 structures form within the cell and mediate alternative events (Juranek and Paeschke 2012; Spiegel et al. 2020). Because of their high stability, the formation of G4 structures needs to be tightly regulated. Misregulated G4 structures or G4 structures formed at the wrong time or location can lead to genome instability. Different experimental approaches have shown that G4 formation can alter transcription, translation and the activity of polymerases and telomerase (Bochman et al. 2012; Rhodes and Lipps 2015; Muellner and Schmidt 2020; Varshney et al. 2020). In summary, misregulated G4 structures lead to a stalled or slowed DNA replication machinery and increase the number of chromosomal mutations, deletions and recombination events (Valton and Prioleau 2016; Bryan 2019; Lerner and Sale 2019). Based on these data, it would be expected that these sequences disappear during evolution. However, the situation is the opposite. During the evolution, the amount of potentially G4-forming sequences increased and the regions that could form G4 structures are more evolutionary conserved than neighbouring regions (Nakken et al. 2009; Capra et al. 2010; Marsico et al. 2019). This indicates a positive function of G4 structures at these regions, most likely in fine-tuning of cellular processes. To counteract the negative genome instability effects, but still benefit from the positive regulatory potential of G4 structures, cells must have developed machinery to control G4 structure formation.

## DNA helicases are needed for G4 replication

In the past years, several different G4-unwinding helicases have been identified (Mendoza et al. 2016; Sauer and Paeschke 2017). They differ from each other based on their directionality as well as their processivity at G4 structures. It is interesting to note that although these helicases unwind G4 structures in vitro they are specific for only a certain set of G4 structures in vivo. It is not clear, yet, how they gain specificity for specific target G4 structures. In *Saccharomyces cerevisiae* at least three DNA helicases can unwind G4 structures in vitro (Pif1, Sgs1 and Hrq1) and have been implicated to function at G4 regions in vivo (Sun et al. 1999; Ribeyre et al. 2009; Piazza et al. 2010; Paeschke et al. 2011, 2013; Byrd and Raney 2015; Hou et al. 2015; Rogers et al. 2017; Dahan et al. 2018; Sparks et al. 2019). Pif1 seems to be the primary G4-unwinding helicase in yeast (Ribeyre et al. 2009; Paeschke et al. 2013). Pif1 is a highly conserved 5′–3′ DNA helicase, which belongs to the SF1 superfamily (Bochman et al. 2010). Pif1 has a mitochondrial and a nuclear isoform (Foury and Dyck 1985; Schulz and Zakian 1994) and multiple functions in the cell. All of these functions are linked to the preservation of genome stability: (I.) Pif1 activity is essential for the maintenance of the mitochondrial genome (Foury and Dyck 1985), (II.) Pif1 cooperates with proteins of the replication machinery (Dna2 and PCNA) (Budd et al. 2006; Buzovetsky et al. 2017) and supports Okazaki-fragment maturation (Stith et al. 2008; Pike et al. 2009), (III.) Pif1 co-localizes with DNA repair foci and suppresses the accumulation of toxic DNA recombination intermediates (Wagner et al. 2006; Wilson et al. 2013), (IV.) Pif1 maintains the replication fork barrier at the ribosomal DNA loci, (Ivessa et al. 2000), (V.) Pif1 negatively regulates telomerase (Schulz and Zakian 1994; Boule et al. 2005; Phillips et al. 2015) and (VI.) Pif1 is associated with putative G4-forming regions in the yeast genome. Pif1 binds and unwinds G4 structures and supports DNA replication (Paeschke et al. 2011, 2013). The strand specificity of Pif1 is not clear yet, but most likely it can act on both leading and lagging strand template DNA (Lopes et al. 2011; Dahan et al. 2018). It is assumed that Pif1's function is supported by additional proteins. It has been shown that Mms1 supports Pif1-binding to a subset of G4 motifs located on the lagging strand template DNA at replication (Wanzek et al. 2017). Surprisingly, Pif1 can unwind G4 structures in an ATP-dependent and ATP-independent manner (Byrd et al. 2018). The current model of the mechanism of Pif1 function at G4 structures during replication is that Pif1 slides on the single-stranded template DNA in 5′–3′ direction in an ATP-dependent manner and resolves G4 structures as a monomer. After the unfolding of the G4 structure, Pif1 is stalled at the primer-template junction of the replication fork. At this point Pif1 is dimerising and the dimer can efficiently unwind the dsDNA after the junction point. Additionally, Pif1 can re-anneal at the junction point the complementary strand of the G4-forming sequence via its strand-annealing activity, which could be a potential way to prevent the re-formation of the unfolded G4 structure (Galletto and Tomko 2013; Zhou et al. 2014; Duan et al. 2015; Li et al. 2016; Zhang et al. 2016).

## Mgs1 preserves genome stability at the replication fork

*S. cerevisiae* Mgs1 (Maintenance of genome stability 1) is a multifunctional protein which belongs to the conserved AAA^+^ ATPase family (Hishida et al. 2001). The exact biochemical mechanism of its action is not known, but its function in genome maintenance was clearly demonstrated: (I.) Its absence leads to an elevated rate of mitotic recombination (Hishida et al. 2001), (II.) overexpression of Mgs1 results in increased DNA damage sensitivity of yeast cells (UV, HU, and MMS) (Hishida et al. 2001, 2002; Branzei et al. 2002a, b) and (III.) *mgs1* is synthetically lethal with *rad6*Δ and shows a synergistic growth defect with *rad18*Δ (Hishida et al. 2002). This data suggests a Rad18-independent, replication-associated function of Mgs1. Mgs1 might be involved in the rescue of stalled replication forks (Barbour and Xiao 2003; Hishida et al. 2006; Vijeh Motlagh et al. 2006). *Mgs1*Δ* sgs1*Δ yeast cells show a slow-growing phenotype (Branzei et al. 2002a, b), Mgs1 stimulates the activity of the DNA polymerase δ (Branzei et al. 2002a, b) and Mgs1 is required to inhibit a recombination salvage pathway at stalled replication forks (Jimenez-Martin et al. 2020). Additionally, Mgs1 may also act in Okazaki-fragment maturation via stimulation of the Fen1 endonuclease (Kim et al. 2005). Mgs1 has a UBZ domain, located at the N-terminal part of the protein and an ATPase domain at the central region (Lehmann et al. 2020). Mgs1 exhibits a DNA-dependent ATPase and single-strand annealing activity (Hishida et al. 2001). These functions are connected to its ATPase domain. Mgs1 interacts with PCNA and exhibits a preference for the association with polyubiquitylated PCNA (Saugar et al. 2012).

The synthetic lethal phenotype of the *mgs1*Δ *rad6*Δ strain can be rescued by overexpression of Mgs1 lacking the UBZ domain (Saugar et al. 2012). This suggests that the Mgs1-dependent rescue of the stalled fork is independent of the DNA damage tolerance pathway and PCNA ubiquitylation. We recently performed an in vivo yeast-one hybrid screen and identified novel G4 interacting proteins. We identified over 100 protein candidates including Slx9 and Zuo1 (Gotz et al. 2019; De Magis et al. 2020). Their G4-binding was already confirmed in vivo and in vivo. We also identified novel G4 structure-binding candidate proteins. The Y1H has the advantage that the screen is done in vivo—protein folding and G4 formation are not altered because of purification steps or biochemical changes. Among these new proteins was Mgs1. It caught our interest due to its role in DNA replication. We confirmed that Mgs1 specifically binds G4 structures in vivo. The binding affinity to G4 structures was 3-to-10-fold higher compared to unstructured DNA. Although the binding affinity of Mgs1 was specific for G4 DNA, we could not monitor a change in ATPase activity upon G4 structure binding (Zacheja et al. 2020).

The binding specificity of Mgs1 to G4 structures in vitro was the first indication of a possible function at G4 structures also in vivo. It did not answer the questions if, when and why Mgs1 binds to G4 structures in vivo. Previous studies have shown that the timing of binding to G4 structures is particularly important. Slx9 only binds to G4 structures during DNA damaging conditions, whereas Pif1 binds to G4 structures only during S phase (Paeschke et al. 2011; Gotz et al. 2019). Similarly to Pif1, Mgs1 binds to G4 structures in vivo even without the addition of DNA damage (Zacheja et al. 2020). The binding of Mgs1 is even stronger/enriched if G4 structures are stabilized by the G4-stabilizing ligand PhenDC_3_. The association of Mgs1 to the G4 structure depends on the presence of Pif1 but it is independent of Sgs1 (Zacheja et al. 2020). This data suggested that Pif1 and Mgs1 act in the same pathway because Pif1’s function partially supports Mgs1-binding to G4 structures. We built a hypothetical model which describes the function of Mgs1 in the replication of G4 structures in association with Pif1 action, based on the available data. We demonstrated that Mgs1's function at G4 structures is essential for genome stability and that G4 structures that lack Mgs1 (in *mgs1*Δ cells) caused increased GCR, accumulation of γH2A as well as slow growth. We did not observe any alteration in replication fork progression in *mgs1*Δ cells under normal conditions.

**Fig. 1:**
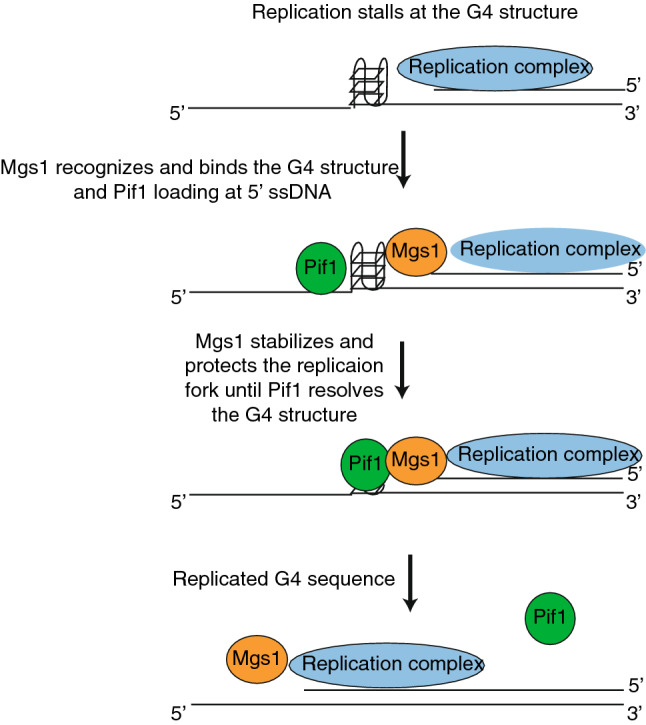
Hypothetical model of the function of Mgs1 at G4 structures. During the replication G4 structures are formed on the single-stranded template DNA, which blocks the replication fork progression and DNA synthesis. Based on our results Mgs1 might be involved in the recognition of G4 structures at stalled replication forks. Binding of Mgs1 stabilizes and protects the replication fork until Pif1 resolves the blocking structure and joins the replication complex. This resumes the movement of the replication fork movement and replication beyond the G4 structure

The current model is that G4 structures, which form during DNA replication, lead to a slowing down of the replication fork as it approaches the G4 structure (Paeschke et al. 2011). Pif1 is recruited and unwinds these G4 structures and suppresses genome instability at these sites (Paeschke et al. 2011). Another work has shown that G4 structures are unfolded or repaired in the next cell cycle (Lemmens et al. 2015). Our data shows that without Mgs1 more DNA damage and genomic rearrangement is observed at G4 structures (Zacheja et al. 2020). We predict that Mgs1 functions to protect the slowed/stalled replication fork at the G4 structures. The major question is how Pif1 binding is connected to Mgs1 binding at G4 structures. We did not observe any direct interaction of Pif1 and Mgs1, but assume that they are functionally connected. Based on published data we predict the following model (Fig. 1): (I.) G4 structures induce stalling of the replication fork, (II.) Mgs1 protects stalled replication forks and anchors them to the G4 structure, (III.) Pif1 resolves G4 structures and interacts with the replication complex via its interaction with PCNA. The unwinding of G4 structures and the re-start of the stalled replication fork stabilizes Mgs1 in the replication complex in concert with Pif1 binding. At this point, we cannot exclude that in the absence of PCNA protein, which is a binding partner of Mgs1 and Pif1, is modified or altered in such a manner that Mgs1 binding is reduced. In summary, we predict that Mgs1 is recruited to G4 structures formed during DNA replication and that its major function is to protect the replication fork and prevent genome instability.

## Further perspectives

How are the intrachromosomal G4 structure-forming sequences replicated? Several questions are still unanswered regarding this process. One of the main questions is the timing of G4-unwinding in wild type cells. Can the normal replication apparatus handle this situation or do all formed G4 structures lead to replication fork stalling? If stalling of the replication fork is induced at every formed G4 structure, the rescue of the stalled replication fork must depend on PCNA ubiquitylation; this process is not examined, yet. Answering this question could also bring us closer to understand the involvement of the different DNA polymerases in intrachromosomal G4 replication. However, the exact biochemical mechanism of Mgs1's function is still an open question. Identification of the amino acid residues of Mgs1 involved in G4-binding would be important to allow deeper analysis of Mgs1 function at the G4 structure. Analysis of genetic interactions between *mgs1* and *pif1* in non-G4 structure-associated pathways would also be important to understand their connection.
